# The Electrophysiology of Atrial Fibrillation: From Basic Mechanisms to Catheter Ablation

**DOI:** 10.1155/2021/4109269

**Published:** 2021-06-05

**Authors:** Panagiotis Ioannidis, Theodoros Zografos, Evangelia Christoforatou, Konstantinos Kouvelas, Andreas Tsoumeleas, Charalambos Vassilopoulos

**Affiliations:** Heart Rhythm Center, Athens Bioclinic, Athens, Greece

## Abstract

The electrophysiology of atrial fibrillation (AF) has always been a deep mystery in understanding this complex arrhythmia. The pathophysiological mechanisms of AF are complex and often remain unclear despite extensive research. Therefore, the implementation of basic science knowledge to clinical practice is challenging. After more than 20 years, pulmonary vein isolation (PVI) remains the cornerstone ablation strategy for maintaining the sinus rhythm (SR). However, there is no doubt that, in many cases, especially in persistent and long-standing persistent AF, PVI is not enough, and eventually, the restoration of SR occurs after additional intervention in the rest of the atrial myocardium. Substrate mapping is a modern challenge as it can reveal focal sources or rotational activities that may be responsible for maintaining AF. Whether these areas are actually the cause of the AF maintenance is unknown. If this really happens, then the targeted ablation may be the solution; otherwise, more rough techniques such as atrial compartmentalization may prove to be more effective. In this article, we attempt a broad review of the known pathophysiological mechanisms of AF, and we present the recent efforts of advanced technology initially to reveal the electrical impulse during AF and then to intervene effectively with ablation.

## 1. Introduction

The need for electrophysiological interpretation of atrial fibrillation (AF) is a perpetual challenge. In recent years, there has been tremendous progress in understanding the pathophysiological mechanisms of AF with the gradual analysis of the seemingly chaotic fibrillatory rhythm. Many times, on this long scientific journey, empirical therapeutic intervention preceded and the recognition or the supposition of the underlying mechanism followed. One of the most basic clinical conclusions, which has been demonstrated in practice by numerous clinical studies, is that the wide and, as much as possible, permanent pulmonary vein isolation (PVI) is the cornerstone for maintaining the sinus rhythm (SR). However, the observation that AF, especially the persistent clinical type, can be maintained in patients with electrically isolated PVs directly raises the question of what should be the next step in the ablation process. In this article, we attempt to review the basic AF electrophysiological mechanisms and the treatment options in catheter ablation.

### 1.1. Historical Background

The first hypothesis about the mechanism of AF began in the early twentieth century, when Sir Thomas Lewis, having the electrocardiogram and the venous pulse recording as the only observational tools, suggested that AF was more complicated than the circus movement of atrial flutter [1]. In 1959, Moe described the multiple wavelet hypothesis in which he extended the theory of reentry to include multiple circuits that occur simultaneously and are self-sustaining [2]. According to this theory, the maintenance of AF is achieved due to the presence of a critical number of reentrant wavelets in the atria, which must have a sufficient mass of myocardium and special characteristics in terms of conduction velocity and refractory period. The multiple wavelet hypothesis was further supported by the application of a virtual computational AF model in which the continuous motion of the wavelets (15 to 30 according to the model) showed self-preservation properties in a similar way as in the clinical AF [3]. Moe et al. showed in this model that the probability of simultaneous cessation of all the wavelets is low and that this fact contributes to the self-preservation of AF. If the number of the wavelets becomes less than a critical limit, then there is a greater chance of their simultaneous disappearance and, consequently, the AF termination. Several years later, Allessie et al. provided experimental data supporting the multiple wavelet theory [4]. In isolated dog hearts, using a Langendorff preparation, it was shown with simultaneous recording from 192 points in the atrial myocardium that the AF could be maintained by 3–6 wavelets rotating simultaneously. More supportive evidence in this theory was provided by experimental evidence in similar models, where it appeared that by administering antiarrhythmic drugs, the termination of AF was facilitated by the reduction in the number of reentrant wavelets [5, 6]. Cox et al. mapped similar electrical activity during human heart surgery, laying the theoretical basis for performing Cox-maze surgery, which had excellent results in AF prevention [7]. It was then widely accepted that multiple mechanisms could play a role in initiating and maintaining AF. Triggers, especially from PVs, can initiate AF and a vulnerable substrate can maintain arrhythmia [8]. Furthermore, as AF is maintained, through the process of remodeling electrical and structural changes in the atrial myocardium can alter the responsible pathophysiological mechanisms [9].

### 1.2. Functional Reentry and the Leading Circle Model

**Figure 1:**
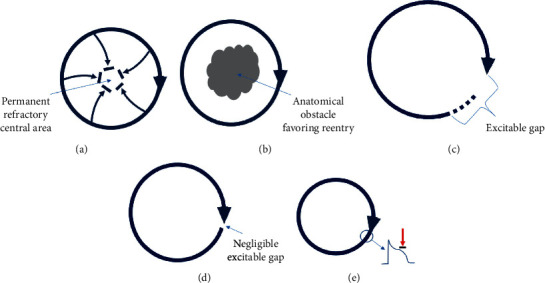
(a) Leading circle reentry. The impulse spreads to the inside of the circuit continuously, making it permanently unexcitable. (b) Typical reentrant circuit around an anatomical barrier. (c) Reentrant circuit with the excitation gap. (d) Reentrant circuit without the excitation gap. (e) Unsustainable reentrant circuit as its frontal part collides with its nonexcitable terminal part.

The simplest form of the functional reentry can be described by the leading circle model, which was first introduced by the French electrophysiologist Allessie in 1977 [10]. According to this model, there is a continuous rotation of the impulse in the periphery of a cycle with simultaneous propagation both peripherally and toward the center of the circle. The impulses directed to the center collide with each other at the same time, making this area refractory. In this way, the impulse is constantly moving around a central, functionally refractory area. This central region in the leading circle concept is the analog of an anatomical barrier (e.g., a scar) which, as we know, can produce a typical, anatomical reentrant circuit. In the typical reentry, the time of a complete rotation is usually longer than the refractory period of the myocardial cells in that area. This means that there is an excitable gap, that is, a time window in which the impulse can enter the circuit finding excitable tissues. If the time of a complete rotation is equal to the refractory period, there is no excitation gap. In functional reentrant circuit such as that of the leading circle, by definition, there is no excitation gap. If the rotation time becomes shorter than the refractory period, then the head of the wavefront will collide into its refractory tail, and the circuit will be terminated (Figure 1). Thus, a functional reentrant circuit without an excitable gap occupies the smallest possible space and rotates at the highest possible frequency, suppressing other potential circuits while having self-sustaining properties.

The multiple wavelets hypothesis and the leading circle concept are complementary, as there is a direct correlation between the cycle length of each circuit, the area it occupies, and the size of the atria. Large atria and reentrant circuits in areas with a short refractory period increase the chance of AF induction and maintenance. In contrast, atria that are small or have been compartmentalized by ablation or maze surgery cannot easily maintain AF. These theories are also confirmed by clinical observations showing AF increases in patients with left atrial dilatation [11], interatrial and intra-atrial conduction disorders [12, 13], and pathologically short refractory periods [14]. However, as can be seen from experimental data, the leading circle model is a simplified approach to the complexity of AF maintenance mechanisms.

### 1.3. The Drivers of AF

Although AF appears to be a chaotic and disorganized rhythm, mapping studies have shown that there are areas with high-frequency depolarization that can be considered as drivers. In these areas, there is a considerable organization, although their activity may spread to the rest of the atrial myocardium in a chaotic and irregular manner, which is characterized as fibrillatory conduction [15]. Mansour et al. [16] in a Langendorff preparation, from sheep hearts, showed that the depolarization frequency in AF introduced with rapid atrial pacing and acetylcholine is much higher in the left atrium than in the right. Consequently, there is a significant frequency gradient from left to right atrium. Areas of dominant frequency are located in the left atrium and in particular in the posterior wall adjacent the PVs. The frequency of depolarization in the left atrium increased with acetylcholine infusion, apparently due to shortening of the action potential, while remaining unaffected after ablation of the interatrial pathways (Bachmann's bundle and inferoposterior pathway). These data suggest that the driving areas of AF are basically located in the left atrium. Of course, whether or not all high-frequency areas represent AF drivers is something that has not yet been clarified.

### 1.4. PVs and Extra-PV Triggers

**Figure 2:**
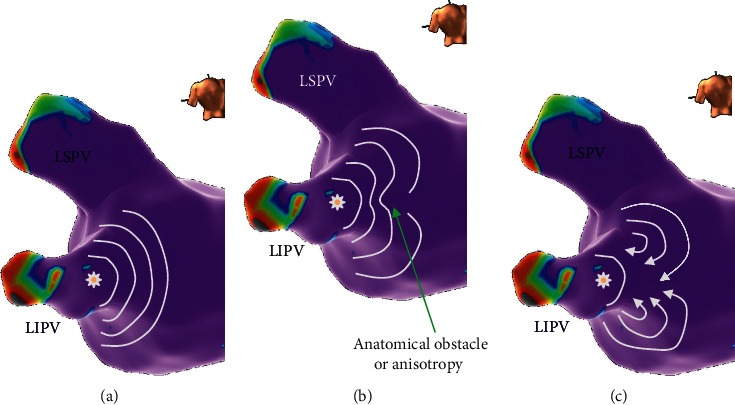
(a) The arrhythmic firing from the left inferior PV propagates normally to the atrial myocardium. (b, c) Special conditions such as conduction anisotropy or anatomical obstacle at the central point of the wavefront can create two waves with rotational propagation, capable of maintaining AF.

The evidence that the dominant frequency is located in the left atrium could be linked with the clinical observation of Michel Haïssaguerre that AF may be initiated by triggers from the PVs [8]. Thus, a significant interest was developed in the pathophysiological role of PVs in AF.  Figure 2 shows the possible mechanism a PV trigger can start rotational activity maintaining AF [17].

There are many reasons for the arrhythmogenic activity of PVs. The PV myocardial cells have a higher concentration of slowly and rapidly activating delayed rectifier potassium channels (IKs and IKr) and a lower concentration of cardiac transient outward potassium current (Ito) and L-type calcium channels than in the rest of the atrial myocardium. This setting is responsible for the hyperpolarization of the resting membrane potential and the shorter duration of the action potential, factors that may favor arrhythmogenesis through reentry and triggered activity [18].

In addition, the anatomical structure in the PVs and the adjacent atrial myocardium appear to play an important role in arrhythmogenesis. The arrangement of the muscle fibers in many layers with circumferential and longitudinal direction, the abrupt change in the orientation of the muscle fibers, and the greater wall thickness in specific positions, such as in the interpulmonary areas and in the ridge between the orifices of the left PVs and the mouth of the left atrial appendage, are some of the anatomical reasons that may promote arrhythmogenesis [19]. In addition, the proximity of the epicardial ganglionated plexi with the PV antra is probably responsible for autonomous modification of the atrial electrophysiological milieu [20]. As shown by the study of Lee et al. [21] in which high-density cardiac mapping was performed during cardiac surgery, the areas of PV-LA junction demonstrate marked functional conduction delay and circuitous activation patterns, creating the substrate for reentry. Many times, in clinical practice, we have seen AF start with an automatic firing from a PV.

**Figure 3:**
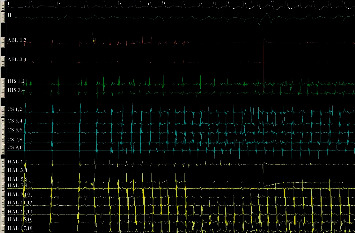
The deflectable 20-polar catheter is placed in the tricuspid annulus in order to perform a linear lesion in the cavotricuspid isthmus. Atrial extrasystoles produced with each movement of the catheters in the right atrium trigger AF.

**Figure 4:**
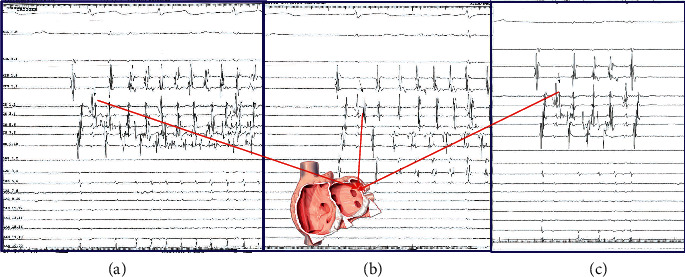
An example of the extrapulmonary trigger. In a patient with long-standing persistent AF, after PVI and the electrical cardioversion to SR, a repeated onset of AF (a, b) is observed with firing from the same point mapped and ablated in the area between left superior PV and LAA (ligament of Marshall). (c) The firing fails to induce AF. Ablation of this area resulted in the long-term SR maintenance for >18 months.

On the other hand, the concept of extra-PV triggers is gaining ground in understanding the mechanism of AF. An extra-PV trigger is an arrhythmic automatic focus, apart from PVs, which with its rapid firing can initiate AF. For the sake of clarity, we must distinguish the concept of the extra-PV trigger from the extra-PV perpetuator. Obviously, the trigger contributes only to AF provocation, while the perpetuator operates during AF supporting its maintenance. Thus, focal or rotational sources outside the area of the PVs occurring during AF cannot be referred to as extra-PV triggers. Probably, a didactic example of a nonclinical extra-PV trigger is the introduction of AF with the extrasystoles caused by a roving catheter in the right atrium (Figure 3). It is often reported that revealing and ablating extra-PV triggers can be done systematically during the procedure and this contributes to SR maintenance [22]. It should be noted that during ablation, the demonstration of an extra-PV trigger that really has a causal role in the onset of AF is a laborious process with many difficulties and limitations. It is a laborious process with many difficulties and limitations. Several provocation protocols such as pharmacological challenges (usually isoproterenol), atrial stimulation, and successive AF cardioversions can be used for the elicitation of these arrhythmic foci [22]. An ectopic focus outside PVs to truly represent an AF trigger must be repetitively exhibited and proven to induce AF (Figure 4). It is unknown whether the mapping and ablation of an ectopic activity that occurs incidentally in SR actually represents a source that could also cause clinical AF. It is also unknown whether and how an arrhythmic focus that appears fleetingly and rarely can be consistently identified during a standard EP study. In this sense, finding areas with characteristics that prove causality in the introduction of AF is rare and difficult. In addition, the lack of repeatability in AF induction makes the process of mapping and exact location of such foci extremely hardworking and time consuming.

### 1.5. Rotors, Spiral Waves, and Fibrillatory Conduction

**Figure 5:**
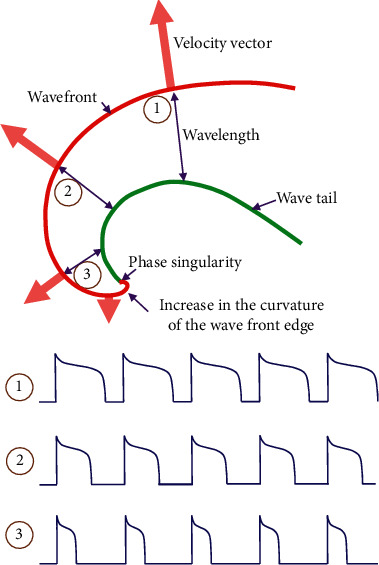
Schematic representation of a rotor. The front of the depolarization wave (red line) propagates at a higher speed in the periphery (point 1) than in the center (point 3). The green line represents the repolarized edge. The wavelength, the refractoriness, and the conduction velocity differ from the periphery to the center; however, the depolarization frequency in a stable rotor is the same (see text for details).

Recent experimental and clinical data describe specific types of functional reentry called rotors or spiral waves. The rotor is characterized by the rotation of a curved depolarization wavefront around a central point (Figure 5). The propagation velocity of the depolarization wavefront is higher at the periphery of the rotor and lower toward the center. Similarly, the cycle length of the depolarization is higher to the circumference as the frontal margin is further away from the tail of the depolarized area, while toward the center, the curvature of the frontal margin increases and its distance from the tail decreases. Thus, the central point of the rotor has a unique frequency of depolarization and is called phase singularity [23]. The phase singularity point is practically unexcitable and its speed is extremely low, but not zero. This fact permits the movement of the central point and therefore of the entire rotor. This characteristic of the rotor distinguishes it from the entirely theoretical model of the leading cycle [24]. The term spiral waves has often been used to describe the same phenomenon as rotors. However, in electrophysiology, rotors are characterized as drivers, while spiral waves refer to the peripheral propagation of rotor-derived impulses. The term scroll waves actually describes the same event in a three-dimensional model [25, 26]. The rotor can be formed when a regular impulse collides at an anatomical barrier (myocardial fibrosis) or a functional barrier (anisotropy).

**Figure 6:**
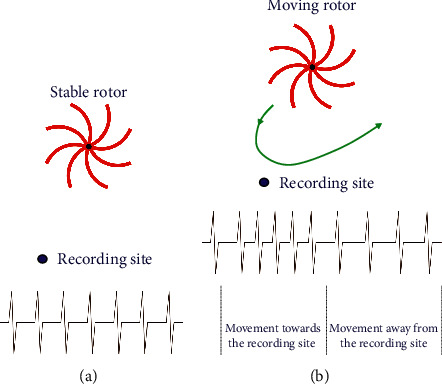
Doppler-type effect with an increase and decrease in the depolarization frequency when the rotor is moving [28]. (a) Stable rotor. (b) Moving rotor.

**Figure 7:**
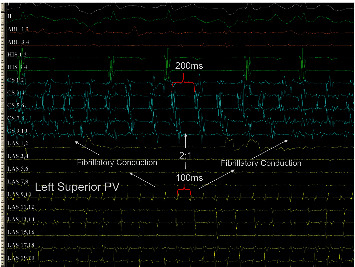
Focal automatic firing (cycle length: 100 ms) from LSPV enters the LA either by 2 : 1 conduction or by fibrillatory conduction.

**Figure 8:**
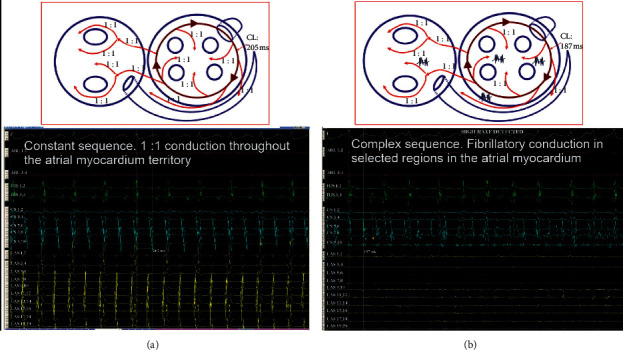
The hypothetical mechanism of the occurrence of fibrillatory conduction in macrocircuits, studied by the technique of selective activation remapping. (a) Clockwise perimitral flutter with 205 ms CL and constant 1 : 1 conduction throughout the atria (regular atrial tachycardia). (b) Acceleration of the CL to 187 ms with fibrillatory conduction (unstable atrial tachycardia) (from Ioannidis et al. [29]).

After its creation, the initial rotor or mother rotor can be broken up into different depolarization waves, which can under appropriate conditions acquire rotor properties, forming the so-called “daughter rotors.” Other times, the waves generated by a rotor can propagate with complete disorganization. This is called fibrillatory conduction [17]. The fibrillatory conduction in the presence of rotors was studied in an AF clinical setting with conventional catheters by Atienza et al. [27] and was found to occur when the depolarization frequency becomes higher. It was assumed that the increase in the frequency is caused by the rotor moving to the recording point, generating a Doppler-type effect (Figure 6). It is clear that any point in the atrial myocardium at a given frequency may exhibit the Wenckebach behavior, that is, an apparent conduction delay responsible for the occurrence of fibrillatory conduction. A rapidly firing arrhythmic focus can be spread to the atrial myocardium exhibiting fibrillatory conduction, possibly creating the conditions for AF induction and maintenance (Figure 7). It is possible that the fibrillatory conduction may be also present during macroreentrant circuits, especially with relatively short cycle length. During sequential mapping of unstable atrial tachycardias, we used the selective activation remapping technique, which provided evidence to support the above hypothesis [29] (Figure 8).

### 1.6. Data from Optical Mapping

In the scientific research of the AF mechanisms beyond theoretical models, conventional mapping, and clinical observations, the optical mapping played a major role. This method sufficiently enriched our knowledge, as we could have high-resolution imaging of the actual electrical impulse during AF. The basic principle of optical mapping is based on the administration of a voltage-sensitive dye, which binds to membrane proteins and has the ability to change its fluorescence when the membrane potential changes during depolarization and repolarization. With appropriately sensitive cameras placed to record the endocardium or the epicardium, this changing in the fluorescence is recorded and so the depolarization and repolarization of the cardiac tissue can be displayed. The obtained recordings undergoing special processing give us a real illustration of the electrical wave propagation [30]. Animal studies have shown that such rotational waves are maintained during AF over an unexpectedly long period of time. The time of a complete rotation, which by definition represents the cycle length, was extremely short, and the frequency of depolarization was extremely high, leading to the reasonable assumption that the cause of the high frequency in the left atrium is the presence of rotors and spiral waves [31].

Another study of optical mapping performed on human hearts places particular emphasis on the importance of reentry circuits to maintain AF. The study was performed on 8 human hearts, with structural heart disease, removed due to transplantation. The area of the lateral wall of the right atrium was excluded and studied with endocardial and epicardial optical mapping. At the same time, high-resolution magnetic resonance imaging was performed on these atrial segments, which could highlight areas with fibrosis and complex orientation of the myocardial fibers. The mapping revealed multiple micro-reentrant circuits, which were located on the entire thickness of the atrial wall, often having an endocardial and cardiac component. The areas where these circuits were found had a higher rate of fibrosis and significant angulation in the orientation of the myocardial fibers. In fact, ablation in these areas could terminate AF and make it noninducible, unlike other control areas in which ablation did not have such an effect [32].

## 2. The Theoretical Knowledge in Clinical Practice

The revelation of rotors and their importance to AF has led to the development of mapping techniques to reveal this electrical activity in clinical practice.

**Figure 9:**
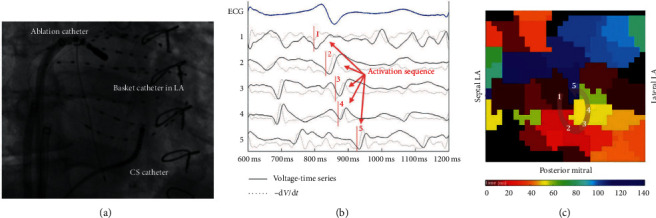
FIRM mapping. (a) The 64-pole basket catheter in the left atrium. (b) ECG (blue) and unipolar intracardiac signals (black) from the 64-pole basket catheter. (c) Isochronal activation map reconstructed from the corresponding electrograms, illustrating a left atrial rotational activity (from Baykaner et al. [51]).

Focal impulse and rotor modulation (FIRM) is an innovating technique for simultaneous AF mapping developed a few years ago. It utilizes a 64-pole basket catheter of 8 splines containing 8 electrodes each that expands to come into contact with the wall of the left or right atrium, while simultaneously mapping the electrical activity during AF. The analysis of the electrical signals can be presented in a video, displaying the activation and the deactivation of a particular point in alternations of white and black color. In this way, the rotational activity can be depicted and subsequently ablated at the corresponding poles of the catheter. This technique, known as FIRM ablation, had very good results in the CONFIRM (Conventional Ablation for Atrial Fibrillation With or Without Focal Impulse and Rotor Modulation) clinical study [33] (Figure 9).

**Figure 10:**
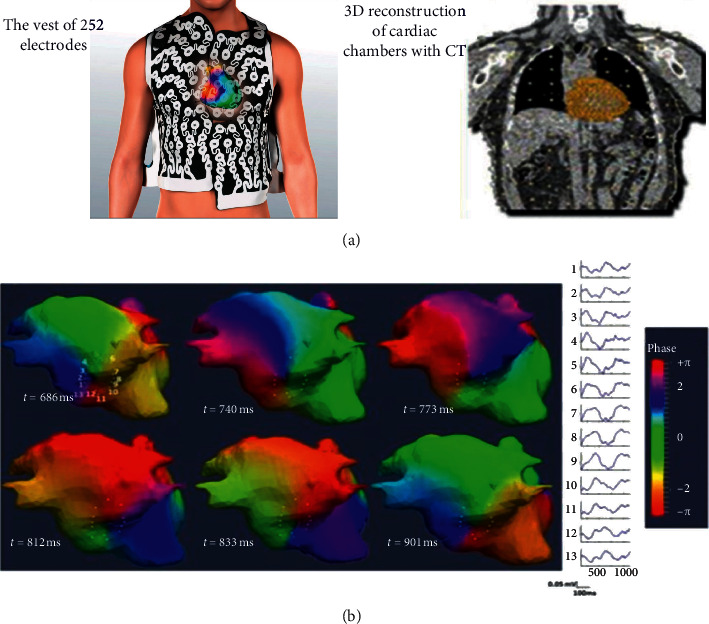
Noninvasive body surface mapping. (a) The vest of 252 electrodes and the three-dimensional reconstruction of cardiac chambers with the computed tomography. The voltage at each point of the torso, taken from the 252 electrodes of the vest, is depicted with virtual electrograms on the surface of the atria. (b) Isochronous maps showing the rotational activity near the antrum of the right inferior PV.

AF mapping was also attempted with the development of another technology with completely different conception which uses noninvasive body surface mapping. By this technology, the electrical activity is recorded by 252 electrodes in the patient's torso, and after computer processing of these recordings, using special mathematical algorithms, the virtual electrical activity is overlaid on the surface of the atria, the three-dimensional model of which has previously been obtained by computed tomography. Thus, we have a simultaneous representation of the electrical activity during AF. Recordings taken with this procedure are also indicative of rotational and focal discharging activity in many areas of both the left and right atrium [34] (Figure 10).

### 2.1. The Anatomical Substrate of Atrial Fibrillation

The development of AF, even in the absence of other comorbidities, may confer several changes in the atrial myocardium, collectively described as structural remodeling. Very short-term AF produces no ultrastructural alterations, while AF lasting several weeks causes primarily cardiomyocyte-dependent changes, and long-term persistent AF produces mixed cardiomyocyte- and fibroblast-dependent changes [35]. These alterations in the atrial cardiomyocytes and the myocardial interstitium have been long known to include chronic inflammatory infiltrates, foci of myocyte necrosis, focal replacement fibrosis, and myocyte cytoplasmic vacuoles consistent with myolysis [36]. The degree of such morphological abnormalities, as cellular hypertrophy and myolysis, has been associated with the presence of AF and atrial dilatation [37]. Atrial dilatation in turn, through an increase in atrial substrate size, has been associated with fibrillatory activity maintenance [38]. Moreover, the resulting fibrosis promotes AF by interrupting fiber bundle continuity and causing local conduction disturbances [39].

In the atrial cardiomyocytes, AF induces changes in the distribution of proteins responsible for intercellular coupling, the connexins. Heterogeneities in connexin-40 distribution may affect anisotropic conduction in the atria and are known to play a pathophysiological role in paroxysmal AF [40]. In animal models, connexin-40 heterogeneity increases with AF duration and is associated with AF stability [41]. Several cardiac diseases associated with AF, such as hypertension, obesity, hyperglycemia, and heart failure, have been shown to affect the normal distribution of connexins in the atrium, thus causing the conduction disturbances needed for sustaining AF [35, 42].

Another common pathway in the development of AF persistence in several AF-promoting conditions is atrial fibrosis. Rapid atrial firing promotes fibroblast differentiation to collagen-secreting myofibroblasts through autocrine and paracrine mechanisms, thereby allowing AF to induce atrial fibrosis [43]. Atrial fibrosis occurs earlier in the course of congestive heart failure (CHF) and to a greater extent than in the ventricles. In animal models, CHF-induced atrial fibrosis has been shown to increase the vulnerability to AF and administration of antifibrotic drugs resulted in a reduction of susceptibility to AF [44]. Other cardiac diseases that may lead to atrial fibrosis include obstructive sleep apnea, hypertension, obesity, diabetes, and valvular heart disease, such as mitral stenosis and mitral regurgitation [45, 46]. In all these cases, the fibrosis has been shown to cause abnormal conduction through the atria, creating a substrate for AF. The mechanism of AF produced by the substrate of atrial fibrosis may include both macroreentry and focal sources that could be originating from RA, LA, or the pulmonary veins [47].

### 2.2. Ablation Techniques and Current Capabilities

Over the years, many ablation techniques have been tested alone or in combination. These included PVI with various means and techniques, the creation of linear lesions [48], with or without confirming bidirectional block, the targeting of complex fractionated atrial electrograms (CFAEs) [49], and the mapping and ablation of ganglionated plexi in order to withdraw the effect of the autonomic nervous system on the genesis of arrhythmia [50, 51]. After this long period of time, nowadays, there is a consensus that the wide PVI must necessarily be the first step in ablation. The reasons for PVI effectiveness are many, but probably the common denominator is the exclusion of sufficient atrial myocardium mass of great pathophysiological importance. So today, with the current technology, we can reliably achieve PVI acutely, without, however, being able to guarantee a permanent result. The recurrence of conduction reconnects the previously isolated critical substrate with the rest atrial myocardium, setting thus the conditions for arrhythmia recurrence and the need for reintervention.

In the last years, significant progress has been made in the technical means regarding PVI. Using contact force catheters and the upgraded cryoballoon, more efficient and therefore more durable lesions were achieved [52–54]. In addition, as the large-scale randomized clinical trial “Fire and Ice” showed, the cryoballoon ablation is as effective as RF least for paroxysmal AF [55]. However, from AF cryoablation series, it has become clear that when acute isolation is more convenient, this can guarantee a better clinical outcome [56]. This observation confirms once again the direct correlation of the clinical effectiveness with the interventional effectiveness in PVI.

One of the important questions in the ablation strategy is which technique is most effective in ablating persistent AF. Since by definition persistent AF has a greater pathophysiological dependence on the substrate, it has been suggested that if more intervention is made, in addition to PVI, better results will be achieved. The STAR-AF II trial [57] examined this question and randomly compared PVI vs. PVI plus CFAE ablation vs. PVI plus linear ablation. It appeared that the arms of the additional ablation did not exceed over the PVI-only strategy. This study has complicated our views on the appropriate ablation strategy in persistent AF. However, this led most of the medical community to adopt the opinion that as the first intervention in cases of persistent AF, only PVI should be attempted.

**Figure 11:**
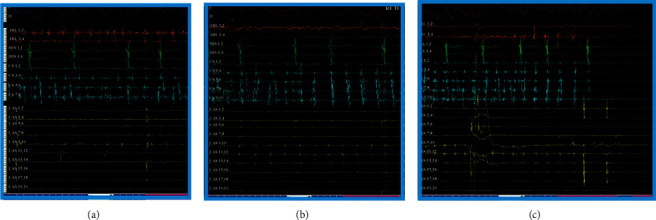
Organization and termination of persistent AF with ablation of CFAEs. (a) Recording of fragmented potentials at the base of the LAA at the onset of ablation. (b) Organization of arrhythmia shortly before termination. (c) Termination of the arrhythmia during ablation in the same area.

However, even if PVs are completely and permanently isolated, the AF can be maintained either because there are extrapulmonary triggers or because the rest of the atrial substrate is so affected that it can maintain the arrhythmia on its own. Making an assessment of our current knowledge in AF ablation, we would say that although we have made great progress in isolating PVs, in localizing pulmonary and extrapulmonary triggers, we have not made much progress in intervening to the substrate. The ablation of CFAEs, subsidiary to PVI, has shown that it can cause organization or termination of arrhythmia [58–60], but nevertheless, it is completely empirical, without knowing in advance whether they are really critical areas or not [61]. In the study of Ammar-Busch et al., CFAEs were indeed correlated with AF drivers as highlighted by noninvasive body surface mapping. It has been found that CFAEs extend to an average of 50% in the atrial surface. Although CFAEs can be detected in 75% of areas with AF drivers, only 25% of CFAEs correspond to areas with drivers. This means that most CFAEs are noncritical areas for maintaining AF. However, through the process of CFAE ablation, we have clinically proven that in the atrial myocardium all areas are not as important in maintaining AF. Some are more important, and targeted intervention on them can terminate or organize the arrhythmia (Figure 11). So came the hypothesis that if we were able to reveal in detail the electrical impulses in AF, we would certainly point out such critical areas susceptible to ablation.

### 2.3. Targeting Drivers and Rotors

Various efforts have been made to reveal the rotational activity during AF. The theoretical background of these efforts is based on the fact that the detection and ablation of rotors, as experimental studies have shown [32], may also have value in clinical practice. FIRM ablation with selective targeting of areas with repeated rotational or focal activity caused termination or organization of KM in 86% of patients with persistent or paroxysmal KM [62]. The initial results of this technique were impressive, as in 9 months of follow-up, in a population with a predominantly persistent (74%) AF, 82.4% of patients remained in SR compared with 44.9% of those who underwent in conventional ablation [33]. In addition, a subanalysis of the CONFIRM trial showed the significance of the elimination of AF rotors and focal sources either directly with system guidance or coincidentally in conventional ablation [63]. However, although the early results of this technique showed impressive results [33, 64–66], more recent studies have failed to confirm the initial encouraging findings [67]. In fact, the recent randomized REAFFIRM trial involving 350 patients with persistent AF showed that FIRM ablation and subsequent PVI are not superior to PVI only [68].

The use of noninvasive body surface mapping has yielded encouraging results, but it is not yet widely used. The rotational activity is illustrated with virtual maps in the epicardium of the atria. Ablation targets areas with repeatable rotational or focal automatic activity. The greatest advantage of this method is that it is noninvasive. On the contrary, the disadvantages include the difficulty of reproducibility and the ability of dynamic mapping during the procedure, as well as the reliability of the provided maps, as the extension is done only in the epicardium of the sinuses and important areas such as the interatrial septum. However, there is also an issue with the reliability of the isochronal maps, as the projection is made only at the epicardium of the atria, and thus important areas, such as the interatrial septum, are practically hidden. In a clinical study of the system, in 103 patients who underwent AF ablation, the targeting of the critical areas terminated AF in 75% of patients with persistent AF and in 15% of patients with long-standing persistent AF. After 12 months' follow-up, 83% of patients with paroxysmal AF and 75% of patients with persistent or long-standing persistent AF remained in SR. The authors claim that the new technique is as effective as the previously used, but that it requires less time [69].

### 2.4. Future Perspectives

**Figure 12:**
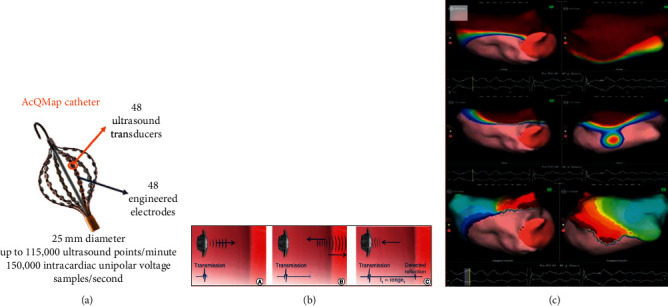
AcQMap noncontact mapping system (Acutus Medical, CA, USA). (a) The multipolar spherical AcQMap catheter with a diameter of 25 mm (deployed), which consists of 6 splines, each of which contains 8 electrodes and 8 ultrasound transducers (total 48). (b) The three-dimensional model of the LA is constructed by emitting ultrasonic waves reflecting them to the cardiac wall. (c) The system displays on the three-dimensional model the propagation of the impulse, calculated by measuring the dipole density.

New technologies are being developed to improve the safety and the efficacy, to reduce fluoroscopy and ablation time, and to provide short-learning curve procedures. In addition, one of the issues that new technologies are called upon to solve is the detailed display of the electrical impulse during AF using simultaneous rather than sequential mapping. The AcQMap mapping system (Acutus Medical, CA, USA) using a 10F multipolar spindle-shaped catheter can create the three-dimensional model of the atrial chamber by emitting ultrasound waves and then display on the reconstructed surface the propagation of the impulse, using data acquired by measuring the charge density [70] (Figure 12). In the UNCOVER AF study, which was conducted in 13 centers in Europe and Canada, on 127 patients with persistent AF, targeting the rotational activities led to 72% maintenance of the SR at 12 months after a single procedure and 93% after 1 or 2 procedures [71].

**Figure 13:**
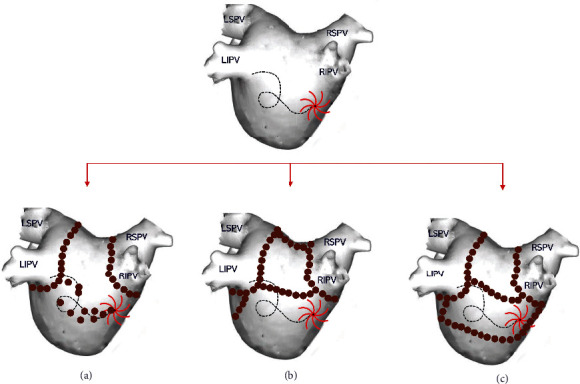
Possible ablation techniques, beyond PVI, after revealing an AF driver. (a) Direct targeting of the rotor path. (b) Standard linear lesions (box lesion and mitral isthmus block). (c) Linear isolation of the rotor path.

A critical question that has not yet been clearly answered is whether the direct targeting of AF drivers, beyond the PVs, can ensure better results. For example, if we target the path of the rotor core, will we be able to prevent AF or in this way are we just trying to catch a cloud? On the opposite side of the spectrum, there is the more rough and less electrophysiological technique of atrial compartmentalization by creating strict areas of block (e.g., standard linear lesions). Obviously, we need more data to decide which of the two approaches is preferable or eventually to be led to a combination of them (Figure 13).

However, in addition to the imaging of the electrophysiological substrate, the detailed imaging of the anatomical substrate would probably be of great help. The location and the extent of atrial fibrosis with late gadolinium enhancement in MRI can give us information that cannot be obtained by any other preinterventional evaluation. The reconstruction of a fibrosis map may guide us in planning the treatment. Nevertheless, from the studies of Marrouche et al. [72], it has been made clear that a high degree of fibrosis is likely a contraindication to conventional ablation strategies, as maintaining SR is achieved at a very low rate in the five-year follow-up [73]. Perhaps, on the other hand, it will not be long before the MRI is systematically performed in the electrophysiological laboratory, while at the same time giving the opportunity to visualize the real effect of the therapy [74].

Until today, even if the ablation efficiency in creating transmural lesions has improved, with the use of contact force and cryoballoon catheters, there are still problems in maintaining PV isolation. A new source of energy tested for AF ablation is the electroporation. By delivering a strong electric field for a very short period of time (<1 ms), through a simple electrophysiology catheter that is in contact with the myocardium, the lipid bilayer of the cell is specifically disrupted. Multiple microscopic pores are formed in the cell membrane, resulting in disturbance of cell homeostasis and apoptosis. The most important feature of electroporation is that the energy threshold that is necessary to create this irreversible damage is different for different tissues. As a result, ablation can be performed in a very short time in the areas of interest of the myocardium, avoiding damage to neighboring organs such as the esophagus [75]. In a recently published study, Reddy et al. using pulsed field ablation with a “one-shot” multispline catheter conducted successful PVI and LA posterior wall isolation in 25 patients with persistent AF [76]. It is obvious that current mapping systems are being prepared for changes in the coming years, as their upgrades will integrate new technologies that have proven to be effective and easy to use. For example, it would be beneficial for all 3D mapping systems to add the ability of one-shot PV ablation. This will save time in relation to the time-consuming point-by-point ablation process and will allow further intervention if needed.

## 3. Conclusions

In an era of rapid developments and changes in the field of AF ablation, it seems that in the coming years the wide PV isolation will remain the cornerstone as ablation strategy. If the new technologies ensure more permanent and safer lesions and allow a detailed depiction of the electrical impulse during AF, then an important step will be taken to definitively treat AF with ablation. The illustration of the electrical activity opens up a new field of research and expectations that we do not know whether it will ultimately be an advantage over current techniques. Of course, we are at the beginning of a journey and the first attempts sometimes have encouraging and sometimes disappointing results. However, the effort to understand the electrophysiology of AF in order to successfully ablate it must not stop, as we seem to be moving toward a more pathophysiological and less empirical approach.

## Data Availability

The data used to support the findings of this study are included within the article.

## Abbreviations

AF: Atrial fibrillation
PV: Pulmonary vein
PVΙ: Pulmonary vein isolation
SR: Sinus rhythm
FIRM: Focal impulse and rotor modulation
CFAEs: Complex fractionated atrial electrograms
LA: Left atrium
CL: Cycle length
LAA: Left atrial appendage
LSPV: Left superior pulmonary vein.
